# A Novel Recombinant Enterovirus Type EV-A89 with Low Epidemic Strength in Xinjiang, China

**DOI:** 10.1038/srep18558

**Published:** 2015-12-21

**Authors:** Qin Fan, Yong Zhang, Lan Hu, Qiang Sun, Hui Cui, Dongmei Yan, Huerxidan Sikandaner, Haishu Tang, Dongyan Wang, Zhen Zhu, Shuangli Zhu, Wenbo Xu

**Affiliations:** 1 WHO WPRO Regional Polio Reference Laboratory and Ministry of Health Key Laboratory for Medical Virology, National Institute for Viral Disease Control and Prevention, Chinese Center for Disease Control and Prevention, Beijing, People’s Republic of China; 2 Xinjiang Uygur Autonomous Region Center for Disease Control and Prevention, Urumqi City, Xinjiang Uygur Autonomous Region, People’s Republic of China

## Abstract

Enterovirus A89 (EV-A89) is a novel member of the EV-A species. To date, only one full-length genome sequence (the prototype strain) has been published. Here, we report the molecular identification and genomic characterization of a Chinese EV-A89 strain, KSYPH-TRMH22F/XJ/CHN/2011, isolated in 2011 from a contact of an acute flaccid paralysis (AFP) patient during AFP case surveillance in Xinjiang China. This was the first report of EV-A89 in China. The *VP1* coding sequence of this strain demonstrated 93.2% nucleotide and 99.3% amino acid identity with the EV-A89 prototype strain. In the *P2* and *P3* regions, the Chinese EV-A89 strain demonstrated markedly higher identity than the prototype strains of EV-A76, EV-A90, and EV-A91, indicating that one or more recombination events between EV-A89 and these EV-A types might have occurred. Long-term evolution of these EV types originated from the same ancestor provides the spatial and temporal circumstances for recombination to occur. An antibody sero-prevalence survey against EV-A89 in two Xinjiang prefectures demonstrated low positive rates and low titres of EV-A89 neutralization antibody, suggesting limited range of transmission and exposure to the population. This study provides a solid foundation for further studies on the biological and pathogenic properties of EV-A89.

Human enterovirus (EV) infections are usually asymptomatic or bring about only mild disease, such as the common cold or minor undifferentiated febrile illnesses. However, EVs are associated with outbreaks of more serious disease such as acute flaccid paralysis (AFP), acute haemorrhagic conjunctivitis, aseptic meningitis, encephalitis, myocarditis, and hand, foot, and mouth disease (HFMD)^1^,^2^,^3^,^4^, which result in considerable morbidity and occasionally in mortality.

EVs belong to the *picornaviridae* family and fall within the new order *Picornavirales,* which represents small non-enveloped RNA viruses with a single stranded positive-sense genome of approximately 7500 nucleotides^5^. The EV genome consists of a single open reading frame (ORF) flanked by 5′ and 3′ untranslated regions (UTRs). The ORF is translated into a single, large polyprotein of 2200 amino acids (aa), which is subsequently cleaved by viral proteases into one capsid protein region (P1) and two non-structural regions (P2 and P3). The P1 region encodes four viral capsid proteins: viral protein 1–4 (VP1–VP4), and the P2 and P3 regions encode seven non-structural proteins 2A–2C and 3A–3D, respectively^6^. The 5′-UTR is about 740 nucleotides long and has an internal ribosome entry site (IRES) that is indispensable for translation initiation^7^,^8^. The approximately 100 nucleotide 3′-UTR, located between the ORF and the poly (A) stretch, forms highly conserved secondary and tertiary structures that are involved in RNA replication^9^.

Currently, more than 100 human EV serotypes have been described. They are currently classified into four species, EV-A, EV-B, EV-C, and EV-D, according to their genomic characteristics^5^,^10^,^11^. The classification of human EVs is based on sequence divergence in the *VP1* coding region, which has been shown to completely correlate with the traditional classification made using antigenic properties^12^. Human EVs can be identified by comparison of the entire or partial *VP1* sequence of an unknown EV to a database of prototype strain sequences. The unknown EV should be classified into the same serotype if they have more than 75% nucleotide identity (85% amino acid identity) in the *VP1* coding region, or into different serotypes if they have less than 70% nucleotide identity (85% amino acid identity) in this region^12^,^13^. However, some isolates may occasionally demonstrate nucleotide identity between 70–75% in the *VP1* coding region, which has been considered a “grey zone” of molecular typing of human EVs. Thus, the use of additional information such as complete *P1* sequence identity for serotype identification may be beneficial for identifying the isolates^14^. The application of molecular typing methods to serologically “untypeable” EV strains has led to the discovery of a large number of new EV types within the four EV species^15^,^16^,^17^,^18^.

To date, species EV-A consists of 21 serotypes including Coxsackievirus 2–8, 10, 12, 14, 16, and EV-A71, as well as the new EV types EV-A76, EV-A89–A92, EV-A114, and EV-A119–A121^19^,^20^,^21^. EV-A89 is a newly identified serotype within the EV-A species. The prototype strain of EV-A89 (strain BAN00-10359/BAN/2000) was isolated from stool specimens of an AFP patient in Bangladesh in 2000^19^. Subsequently, several other EV-A89 strains were isolated from AFP patients, acute gastroenteritis patients, or healthy individuals during disease surveillance activities (such as AFP case surveillance) in Bangladesh^19^,^22^, India^23^,^24^,^25^,^26^, and Egypt^27^. Currently, only one full-length genome sequence (the EV-A89 prototype strain) is available in the GenBank database. Besides the prototype strain, six entire *VP1* sequences and several partial *VP1* sequences of EV-A89 strains are available in the GenBank database. However, no EV-A89 sequences have been reported in China.

In this study, we report the molecular identification and genomic characterization of an EV-A89 strain (strain KSYPH-TRMH22F/XJ/CHN/2011, hereafter referred as strain KSYPH-TRMH22F) isolated in 2011 from a contact of an AFP patient during AFP case surveillance in Xinjiang Uygur Autonomous region of China. This was the first report of EV-A89 in China. Its genetic characteristics and phylogenetic relationship to other EV strains were also investigated.

## Results

### Isolation and molecular typing of the Chinese EV-A89 strain

The virus grew only in the RD cell line. Cell cultures were harvested after a complete EV-like cytopathic effect (CPE) was observed. We determined the partial *VP1* coding region sequence of the KSYPH-TRMH22F strain, which was amplified by primer pairs 486 and 488^28^. Molecular typing of this strain based on the partial *VP1* sequence was then performed using an online EV genotyping tool^29^. The results indicated that the KSYPH-TRMH22F strain belongs to the EV-A89 serotype.

### Complete genome sequence of the Chinese EV-A89 strain

The complete genome of the Chinese EV-A89 strain was determined. It was 7429 nucleotides in length, including a 5′-UTR of 747 nucleotides, a single ORF of 6588 nucleotides encoding a single polyprotein of 2195 amino acids, and a 3′-UTR of 94 nucleotides preceding the poly (A) stretch. Compared to the EV-A89 prototype strain (BAN00-10359/BAN/2000), no nucleotide insertions or deletions were observed in the whole genome. The overall base composition of the KSYPH-TRMH22F strain was 29.56% A, 22.43% C, 22.05% G, and 25.97% U.

A comprehensive comparison of the nucleotide sequence and deduced amino acid sequence of the Chinese EV-A89 strain with the EV-A89 prototype strain and other prototype strains belonging to EV-A is shown in Table 1. Overall, the complete genome sequence identity and the deduced amino acid sequence identity between the Chinese EV-A89 strain and the EV-A89 prototype strain were 91.7% and 98.3%, respectively. The *VP1* coding sequence of this strain showed 93.2% nucleotide and 99.3% amino acid identity with the EV-A89 prototype strain, and 90.0–92.6% nucleotide and 98.6–98.9% amino acid identity with the other six reported EV-A89 strains available in the GenBank database. However, it had 55.7–68.7% nucleotide and 53.7–74.6% amino acid identity with the prototype strains of other EV-A serotypes, confirming that it belongs to the EV-A89 serotype, based on the molecular typing criteria^30^.

The 3′-UTR of both the Chinese EV-A89 strain and the EV-A89 prototype strain contain 94 nucleotides preceding the poly (A) stretch, which is the same length as EV-A76, EV-A90, and EV-A91. Further comparison based on this region revealed that the Chinese EV-A89 strain had 91.4% nucleotide identity with the EV-A89 prototype strain. Compared to the 3′-UTR sequences of the prototype strains of the EV-A76, EV-A90, and EV-A91, the Chinese strain had 88.2%, 94.6%, and 96.8% nucleotide identity, respectively. The Chinese strain demonstrates an obvious difference from the other EV-A types, which had only 30.3–59.4% nucleotide identity in the 3′-UTR.

### Phylogenetic analysis of the Chinese EV-A89 strain and other EV-A prototype strains

**Figure 1:**
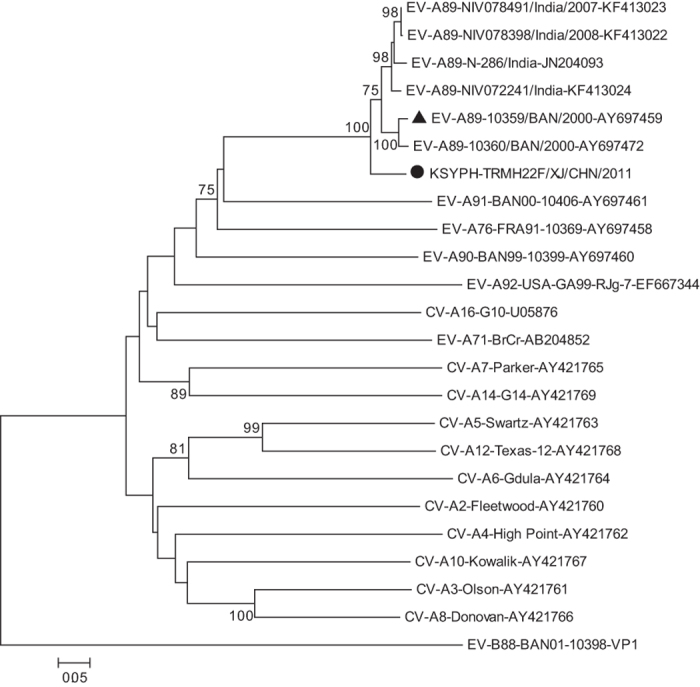
Phylogenetic relationships based on partial *VP1* genomic sequences of enterovirus A89 (EV-A89). The Chinese EV-A89 strain isolated in this study (solid circles), other EV-A89 strains (available in the GenBank database), and other EV-A prototype strains were analysed based on the 888nt (nucleotide 2452–3339) entire *VP1* coding region sequence. The triangle represents the EV-A89 prototype strain. The EV-B88 prototype strain served as an out-group.

Entire or partial *VP1* nucleotide sequence analysis can be used to investigate the phylogenetic relationship among human EVs, however entire *VP1* sequence analysis can provide more information^12^. Therefore, phylogenetic trees were generated from the 888nt (nucleotide 2452–3339) entire *VP1* coding region of the Chinese EV-A89 strain and six other EV-A89 strains available in the GenBank database (Fig. 1). The Chinese EV-A89 strain segregates together with Bangladesh EV-A89 strains (including EV-A89 prototype strain) and India EV-A89 strains, and is monophyletic in comparison, with a longer genetic distance. This suggests a long evolution time and a high degree of genetic divergence among these EV-A89 strains.

**Figure 2:**
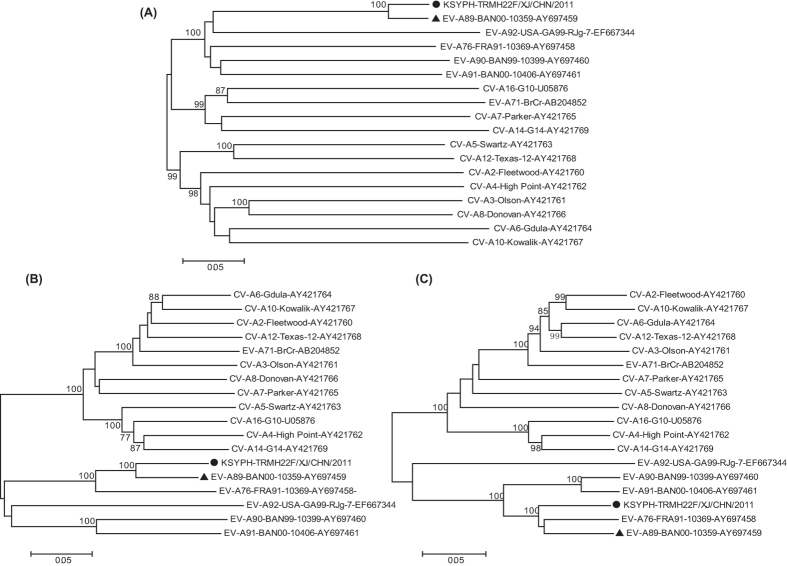
Phylogenetic relationships based on the *P1, P2*, and *P3* genome regions of enterovirus A (EV-A). The Chinese EV-A89 strain (solid circles) and 17 EV-A prototype strains were analysed by nucleotide sequence alignment using the neighbour-joining algorithms implemented in the MEGA 5.0 program. Numbers at the nodes indicate bootstrap support for that node (percentage of 1000 bootstrap replicates). The solid triangle indicates the EV-A89 prototype strain. The scale bars represent the genetic distance. All panels have the same scale. (**A**) *P1* coding sequences, (**B**) *P2* coding sequences, and (**C**) *P3* coding sequences.

Moreover, we constructed phylogenetic trees based on the entire *P1, P2*, and *P3* regions of the genome. In the *P1* capsid coding regions, the Chinese EV-A89 strain clustered together with the EV-A89 prototype strain, with a bootstrap value of 100 (Fig. 2A), thus confirming the preliminary molecular typing results. The phylogenetic trees also showed considerable difference in the non-capsid coding regions. In the *P2* and *P3* coding regions, the Chinese EV-A89 strain shared a distinct high identity with the EV-A76, EV-A90, and EV-A91 prototype strains (Fig. 2B,C). The phylogenetic analysis indicated that one or more potential recombination events between EV-A89 and the three EV-A serotypes might have occurred.

### Recombinant structure of the Chinese EV-A89 strain

**Figure 3:**
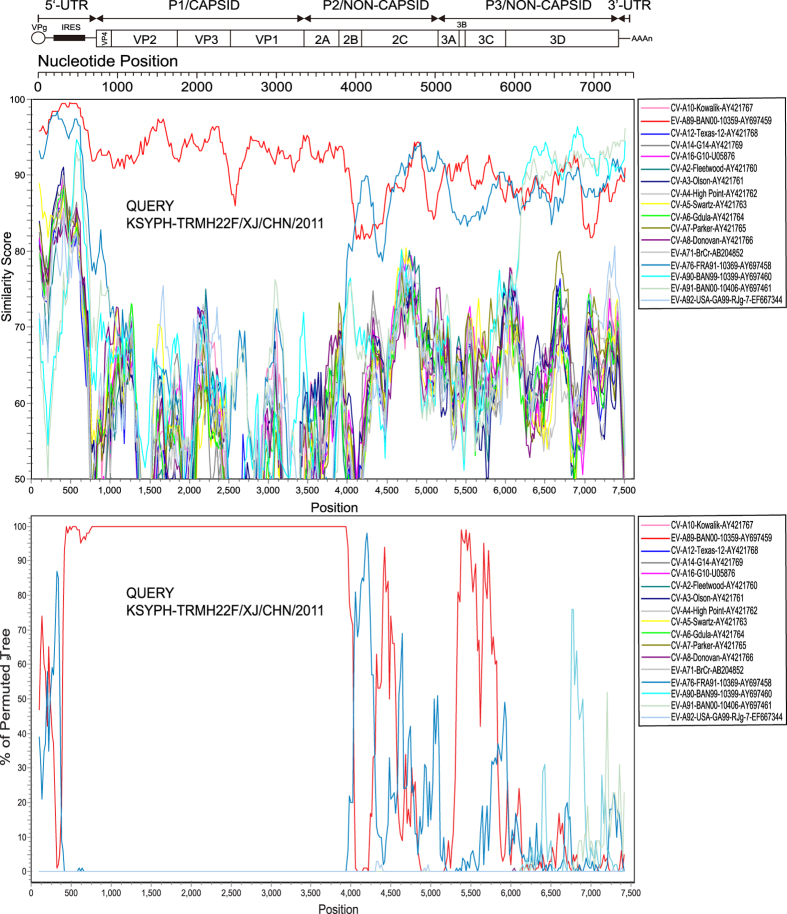
Recombination analyses of complete enterovirus A (*EV-A*) genomes. (a) Similarity plot and (b) bootscanning analysis. A sliding window of 200 nucleotides was used, moving in 20-nucleotide steps. The Chinese EV-A89 strain KSYPH-TRMH22F/XJ/CHN/2011 was used as a query sequence.

Similarity plot and bootscanning analyses were performed to confirm the existence of recombination events between the Chinese EV-A89 strain and the other EV-A prototype strains (Fig. 3). The Chinese EV-A89 strain was used as a query sequence and was compared to the EV-A89 prototype strain and other EV-A prototype strains. In the *P1* and partial *P2* (*2A* and *2B*) coding regions, the Chinese EV-A89 strain demonstrated more than 80% identity with the EV-A89 prototype strain, reflecting a close relationship. However, in the *2C* and partial *P3* regions, the sequence similarities with the EV-A76, EV-A90, and EV-A91 prototype strains were relatively higher. Bootscanning analysis revealed the existence of recombination events between the Chinese EV-A89 strain and the EV-A76, EV-A90, and EV-A91 strains.

### Sero-prevalence of EV-A89 in Xinjiang of China

A total of 60 serum samples were collected from infants and children between 0 months and 4 years of age in the place where the virus was isolated in Xinjiang. Among these 60 serum samples surveyed, 30 were collected from the Kashgar region and the other 30 were collected from the Hotan region. A micro-neutralization assay against EV-A89 showed that 24 serum samples were positive for EV-A89 (>1:8) with a total positive rate of 40.0%. The geometric mean titre (GMT) was 1:43.97 among the positive sera samples. The composition ratios for the EV-A89 neutralization antibody titres of <1:8, 1:8–1:64, and >1:64 were 60.0%, 28.3%, and 11.7%, respectively (Table 2). However, compared to sero-epidemiology studies of other EVs in China, the positive rate of the EV-A89 neutralization antibody and GMT were relatively lower than that of other EVs such as EV-A71 and CV-A16 in the same age group (0–4 years old)^31^.

Although serum samples from the Kashgar and Hotan regions showed a lower positive rate of EV-A89 neutralization antibody and GMT, there were some differences between the two regions. In the Kashgar region, the positive rate of the EV-A89 neutralization antibody and GMTs were 53.3% and 1:66.8, respectively, while in the Hotan region, they were 26.7% and 1:19, respectively. The positive rate of the EV-A89 neutralization antibody and GMTs in the Kashgar region (in the place where the virus was isolated) were significantly higher than those in the Hotan prefecture (seroprevalence rate: p = 0.035 < 0.05, GMTs: p < 0.0001).

## Discussion

In China, a high-sensitivity AFP case surveillance system to detect the presence of wild polioviruses in a population was established in 1994 as part of the Global Polio Eradication Initiative (PEI), and represents the gold standard surveillance system for PEI^32^. Although there is no special surveillance system to detect new EV types, a certain number of new EV types, known as non-polio EVs, have been isolated during the AFP case surveillance for polioviruses. These new EV types, such as EV-A76, EV-B81, EV-B85, EV-C96 and so on, which have been identified and analyzed using the molecular typing method, can provide valuable information on the molecular epidemiology of local novel EVs^17^,^33^,^34^,^35^. Here, we report the complete genome sequence of a novel EV type, EV-A89, which was identified for the first time in China, and was detected during AFP case surveillance.

The Chinese EV-A89 strain described in this study was isolated in 2011 in the Yopurga prefecture in the Kashgar region, which is located in the southern part of Xinjiang. Xinjiang is a frontier in China and is located in the center of Eurasia, adjacent to some Middle East and South Asian countries. All the EV-A89 strains deposited in the GenBank database to date are distributed in Middle East and South Asian countries, and almost all the EV-A89 strains were isolated from patients with AFP except for a strain from Egypt^27^. Therefore, we speculate that EV-A89 infection may correlate with AFP, although there is not enough evidence to demonstrate this relationship since only a few EV-A89 strains have been discovered worldwide. Therefore, more information and data are needed to reveal the biological and pathological properties of EV-A89.

The phylogenetic trees based on the *P2* and *P3* coding region sequences showed that the Chinese EV-A89 strain is closely related to EV-A76, EV-A90, and EV-A91, suggesting the existence of recombination events in these regions. These four EVs (EV-A76, EV-A89, EV-A90 and EV-A91) may have evolved from the same ancestor due to their similar genomic structures and high nucleotide and amino acid similarities. Particularly, there is high nucleotide identity in the *P2* region, *P3* region, and 3′-UTR between EV-A76 and EV-A89, which implies an evolutionary relationship between these two novel EV types. A recombination analysis of the Chinese EV-A89 strain and other EV-A prototype strains in all regions of the genome demonstrated that, regardless of regions (*P1, P2* or *P3*), the Chinese EV-A89 strain is clustered to a distinct monophyletic subgroup with the EV-A76, EV-A90, and EV-A91 prototype strains, and is distinct from the other EV-A members, indicating that the recombination barrier may exist within EV-A species^19^. Based on the findings of the similarity comparison and recombination analysis, it is reasonable to conclude that long-term evolution of these viruses originated from the same ancestor, which provides the spatial and temporal circumstances for recombination to occur.

With the usage of EV molecular typing method, more and more new EVs types were identified, such as EV-B73^36^, EV-A76^33^, EV-B85^34^, and EV-B106^37^, lots of new EV types are proved to be novel recombination variant, indicated that recombination is a frequent event in EV evolution that usually occurs between viruses of the same species and is correlated with the appearance of new EV lineages or EV types. In this respect, the Chinese EV-A89 strain is no exception.

Previous considerable researches indicated the recombination and mutation among enteroviruses have been generally recognized as the frequent mechanisms and features in the EV evolution, based on a large number of epidemiological investigations^38^,^39^. Due to the absence of proof reading mechanism, the mispairing rate of the viral polymerase of EVs is unusual high, under these circumstances, recombination plays a crucial and high effective role in viral evolution by repairing deleterious mutations in EV genomes, thus frequent recombination contributes to their genetic diversity and provides the ability for large evolutionary transitions, by producing in a single step significantly divergent genomes, better response and adapt to new environmental challenges^40^,^41^. For instance, EV-A71 is the most heavily studied EV-A type due to numerous large HFMD outbreaks with high morbidity and mortality caused by EV-A71 have occurred in Asian countries and regions in recent years, a lot of epidemiological and etiological studies indicated that recombination is a frequent phenomenon and is the main force for the evolution of EV-A71^42^,^43^,^44^,^45^.

Recombination with closely related non–poliovirus EV-C species frequently occurs during wild poliovirus circulation and is an indication of person-to-person transmission, and appears to facilitate the emergence of circulating vaccine-derived polioviruses (cVDPV) by replacement of attenuating sites in a single event. However, recombination with EV-C may not be obligatory for cVDPV emergence especially type 1 VDPV, due to type 1 cVDPV circulating locally in China^46^ and the United States^47^ had non-recombinant genomes. Although the possible role of recombination in the phenotypic drift of vaccine strains to a wild strain is not very clear, we propose that recombination with species EV-C may not be essential and is not correlated with the phenotypic drift to higher neurovirulence and higher transmissibility, but it may be an epidemiology indicator of the duration of viral circulation in the human community.

EV-A89 is a newly identified serotype within the EV-A species. Currently, only one full-length genome sequence (the EV-A89 prototype strain) and 12 other EV-A89 sequences are available in the GenBank database, and this is the first and the only report of EV-A89 in China. Due to the limited number of EV-A89 isolates in China and the world, the EV-A89 epidemiological data is very short, so the biological and pathogenic properties of EV-A89 are currently difficult to study in detail. However, we conducted a small range of sero-epidemiology studies, and our research team is currently using reverse genetic methods to explore the advantages of virus recombination found in the Chinese EV-A89 strain.

To investigate the sero-epidemiology of EV-A89 in Xinjiang China, a sero-epidemiology survey was conducted in the places where the virus was isolated. The results showed a prominent difference in the positive rates of neutralizing antibody and GMTs of EV-A89 between the Kashgar and Hotan regions in Xinjiang, indicating a geographical difference in EV-A89 infection. The seropositive rate of EV-A89 neutralization antibody and GMT were relatively higher in the Kashgar region than in the Hotan region (p < 0.05). This suggests that the EV-A89 strain, KSYPH-TRMH22F, isolated in 2011 in the Yopurga prefecture of the Kashgar region may have circulated in the Kashgar region more widely than in the Hotan region. However, the seropositive rate and GMT of EV-A89 neutralization antibody in the two regions were generally lower than other EVs prevalent in China, such as EV-A71 and CV-A16^31^, suggesting limited range of transmission and exposure of the novel EV type EV-A89 to the population. This is consistent with the low viral isolation rate of EV-A89 strains in China (only one EV-A89 strain has been isolated since 1988), and further research will be required to corroborate these conclusions.

In conclusion, this is the first report of the complete genome sequence of a recently described novel EV type, EV-A89, in China. The extremely rare isolation rate suggests that it has not been a prevalent EV serotype in China or even in the world until now. The Chinese EV-A89 strain is closely related to EV-A76, EV-A90, and EV-A91, suggesting the existence of recombination events in non-structure regions. Long-term evolution of EV-A76, EV-A89, EV-A90, and EV-A91 originated from the same ancestor, in order to provide the spatial and temporal circumstances for recombination to occur. Although the biological and pathogenic properties of EV-A89 are currently difficult to study in detail due to the limited number of EV-A89 isolates, we believe that this study provides a solid foundation for further studies on EV-A89.

## Materials and Methods

### Sample collection

This study did not involve human participants or experimentation. The only human material used was a stool sample collected from a close contact of an AFP patient at the instigation of the Ministry of Health P. R. of China for public health purposes. Written informed consent for the use of clinical samples was obtained from all patients involved in this study. This study was approved by the second session of the Ethics Review Committee of the National Institute for Viral Disease Control and Prevention, Chinese Center for Disease Control and Prevention, and the methods were carried out in accordance with the approved guidelines.

The EV-A89 strain (strain KSYPH-TRMH22F/XJ/CHN/2011) was isolated from a stool sample of an asymptomatic contact residing in the Yopurga prefecture of the Kashgar region, in the southern part of the Xinjiang of China. The sample was collected in 2011 during the course of poliovirus surveillance activities in support of the global PEI.

Sixty healthy children ≤ 5 years of age were surveyed for a seroprevalence study of EV-A89 antibodies. Serum samples were collected randomly in 2013, with informed parental consent, by the Xinjiang Center for Disease Control and Prevention: 30 samples were collected in the Kashgar region in the place where the virus was isolated and 30 were collected in the Hotan prefecture (neighboring Kashgar). None of the participants demonstrated any signs of disease at the time of sample collection.

### Viral isolation

The stool sample from the healthy contact was collected and processed according to the World Health Organization (WHO) laboratory manual^48^. The processed sample was used to inoculate three cell lines for viral isolation: a genetically engineered mouse cell line expressing the human poliovirus receptor (L20B cell), human rhabdomyosarcoma (RD), and human laryngeal epidermoid carcinoma (HEp-2). These cell lines were obtained from the WHO Global Poliovirus Specialized Laboratory USA, but were originally purchased from the American Type Culture Collection.

### Molecular typing

The viral RNA was extracted from the viral isolates using a QIAamp Viral RNA Mini Kit (Qiagen, USA) according to the manufacturer’s instructions, and stored at −80 °C.Primer pairs 486 and 488 were used for amplifying the partial *VP1* coding region^28^. RT-PCR (reverse transcription–polymerase chain reaction) was performed using the PrimeScript One Step RT-PCR Kit Ver.2 (TaKaRa, Dalian, China) as previously described^17^. Amplicons were purified using the QIAquick PCR purification kit (Qiagen, Germany) and sequenced using an ABI 3130 Genetic Analyser (Applied Biosystems, Hitachi, Japan). Every nucleotide position of each strand was sequenced at least once. The obtained VP1 sequences were compared with sequences available in the GenBank database for molecular typing using the EV Genotyping Tool^29^.

### Whole genomic sequencing

The 5′ end sequence of the genome was obtained using a 5′ rapid amplification of cDNA ends (RACE) core set (Takara Biomedicals) according to the manufacturer’s instructions. The 3′ end sequence of the genome was obtained by amplification using an oligo-dT primer (primer 7500A)^49^ as the downstream primer. The rest of the genome was determined using the primer walking method. The primers used for PCR amplification and sequencing are listed in Table 3.

### Phylogenetic trees and bioinformatics analyses

The nucleotide and deduced amino acid sequences of the Chinese EV-A89 strain were compared with those of the prototype EV-A strains by pairwise alignment using the MEGA program (version 5.03)^50^. Phylogenetic trees were constructed by the neighbour-joining method, implemented in the MEGA program, using the Kimura 2-parameter model. Bootstrapping was performed with 1000 bootstrap replicates and bootstrap values greater than 80% were considered statistically significant for grouping. Similarity plots and bootscanning analyses were performed using the SimPlot 3.5.1 program. A 200-nucleotide window was moved in 20-nucleotide steps and bootscanning analyses were operated using the neighbour-joining method^51^.

### Neutralization tests against EV-A89

In this study, neutralizing antibodies against EV-A89 were detected using the micro-neutralization assay using the human RD cell line as previously described, with some modifications^31^. Serum samples were inactivated at 56 °C for 30 min before use, and were serially diluted from 1:4 to 1:1024.Virus samples (50 μL) with a tissue culture infective dose (TCID_50_) of 100 were then mixed with the appropriate serum dilution (50 μL), added to a 96-well microplate, and incubated at 37 °C for 2 hours. RD cells (2 × 10^5^ cells/ml) were then added, and the mixture was further incubated at 36 °C in a CO_2_ incubator for 7 days. Virus back titration was simultaneously performed to verify whether the micro-neutralization assay was effective (virus back titration values between 32 and 320 TCID_50_/well indicated that the test was effective). The highest dilution of serum that protected 50% of the cultures, based on the observation of EV-like CPE, was recorded. The serum sample was considered positive if the neutralization antibody titre was 1:8. The GMT was then calculated for positive samples.

### Statistical analysis

Statistical analyses were carried out using the SPSS version 13.0 software (SPSS Inc., Chicago, IL, USA). A chi-square test was used to compare the sero-prevalence rates of EV-A89 between the Kashgar and Hotan prefectures, and a Mann-Whitney test was used to determine the significance of EV-A89 GMTs. Titres below 1:8 were assumed to be 1:4 for calculation. An error value of p < 0.05 indicated statistical significance.

### Nucleotide sequence accession numbers

The complete genomic sequence of the EV-A89 strains (KSYPH-TRMH22F) described in this study was deposited in the GenBank database under the accession number KT277550.

## Additional Information

**How to cite this article**: Fan, Q. *et al.* A Novel Recombinant Enterovirus Type EV-A89 with Low Epidemic Strength in Xinjiang, China. *Sci. Rep.* **5**, 18558; doi: 10.1038/srep18558 (2015).

## Figures and Tables

**Table 1:** The nucleotide sequence and deduced amino acid sequence identities of the Chinese EV-A89 strain KSYPH-TRMH22F with the EV-A89 prototype strain (BAN00-10359/BAN/2000) and other prototype strains belongs to EV-A types.

Region	% nucleotide identity (%amino acid identity)			
	Identity with BAN00-10359 (%)		Identity with other EV-A(%)	
	Nucleotide	Amino acid	Nucleotide	Amino acid
5′-UTR	99.6		72.1–93.7	
VP4	93.7	100.0	61.8–84.0	65.2–100.0
VP2	93.9	99.2	65.8–71.8	73.7–82.5
VP3	93.8	99.5	66.3–71.1	71.7–84.3
VP1	93.2	99.3	55.7–68.7	53.7–74.6
2A	93.3	98.6	66.0–72.0	70.0–81.3
2B	87.5	98.9	62.2–85.1	73.7–97.9
2C	88.6	99.3	73.8–89.9	83.8–99.0
3A	90.6	100.0	68.6–85.6	63.9–100.0
3B	90.0	95.4	60.6–84.8	77.2–95.4
3C	90.1	98.9	71.9–88.1	80.8–99.4
3D	88.8	98.2	72.2–92.4	83.1–98.7
3′-UTR	91.4		30.3–96.8	

**Table 2:** The composition ratios for the EV-A89 neutralization antibody titers.

Titers	Kashgar prefecture		Hotan prefecture		Total(%)
	Number of samples	Ratio(%)	Number of samples	Ratio(%)	
<1:8	14	46.7	22	73.3	36(60.0)
1:8–1:64	9	30.0	8	26.7	17(28.3)
>1:64	7	23.3	0	0	7(11.7)
Total	30		30		60

**Table 3:** PCR and sequencing primer.

Primer	Nucleotide position (nt)	Primer sequence (5′-3′)	Orientation	Reference
0001S48		GGGGACAAGTTTGTACAAAAAAGCAGGCTTTAAAACAGCTCTGGGGTT	Forward	49
EV89-874A	854-874	TAGTGGCCGAAGCTGCGTATG	Reverse	This study
EVP4	541-560	CTACTTTGGGTGTCCGTGTT	Forward	52
0L68-1	1178-1197	GGTAAYTTCCACCACCANCC	Reverse	52
EV89-982S	982-1000	AGTGACAGAGTGGCGCAAC	Forward	This study
EV89-2470A	2451-2470	TGTCTTCCATGGGGTCACCT	Reverse	This study
E486	2297–2322	TGGTAICARACIAAITWYGTIGTNCC	Forward	28
E488	3063–3038	GTIGGRTAICCITCITARAACCAYTG	Reverse	28
EV89-3521A	3502-3521	CCGGTTGTGCACTGACATCT	Reverse	This study
EUG3a	3002-3021	TGGCAAACTTCCWCCAACCC	Forward	53
EUC2b	4469-4488	GGTTCAATACGGTGTTTGCT	Reverse	53
EV89-3827S	3827-3846	TTGGCACAG GAT TCA CAGAC	Forward	This study
EV89-4873A	4854-4873	CATCCAATCTTCCAGACTCC	Reverse	This study
EV89-4716S	4716-4735	TGTCTCCTTCACCTCTAAGT	Forward	This study
EV89-5897A	5878-5897	CTGCCATTCCCTCCAATGTG	Reverse	This study
EV89-5412S	5412-5431	CTTTGCCCTTTCCCTACTCA	Forward	This study
EV89-6256A	6237-6256	TTCCATAACAAGCATCCTCC	Reverse	This study
EV89-6001S	6001-6020	GGCCCAACCAAGACTAAACT	Forward	This study
EV89-6962A	6943-6962	ATAGGGAAGGGGTAACTAGC	Reverse	This study
EV89-6789S	6789-6808	CTGTGTAATGGGCGGAATGC	Forward	This study
7500A		GGGGACCACTTTGTACAAGAAAGCTGGG(T)_24_	Reverse	49
